# The Distribution and Health Risk Assessment of Metals in Soils in the Vicinity of Industrial Sites in Dongguan, China

**DOI:** 10.3390/ijerph13080832

**Published:** 2016-08-19

**Authors:** Chao Liu, Liwen Lu, Ting Huang, Yalin Huang, Lei Ding, Weituo Zhao

**Affiliations:** 1 School of Public Administration, China University of Geosciences, 388 Lumo Road, Wuhan 430074, China; dxy2006@cug.edu.cn (C.L.); luliwen1009@163.com (L.L.); aylinhuang@163.com (Y.H.); 2 Laboratory of Geographical Environment and National Park, China University of Geosciences, 388 Lumo Road, Wuhan 430074, China; 3 School of Environmental Studies, China University of Geosciences, 388 Lumo Road, Wuhan 430074, China; 13476812887@163.com; 4 The Center of Environmental Engineering and Assessment, No. 203 Research Institute of Nuclear Industry, Xianyang 712000, China

**Keywords:** contaminated soil, geoaccumulation index, metal, pollution indexes, human exposure

## Abstract

Exponential industrialization and rapid urbanization have resulted in contamination of soil by metals from anthropogenic sources in Dongguan, China. The aims of this research were to determine the concentration and distribution of various metals (arsenic (As), cadmium (Cd), chromium (Cr), copper (Cu), mercury (Hg), nickel (Ni), lead (Pb) and zinc (Zn)) in soils and identify their potential health risks for local residents. A total of 106 soil samples were collected from the vicinity of industrial sites in Dongguan. Two types of samples were collected from each site: topsoil (0–20 cm, TS) and shallow soil (20–50 cm, SS). Results showed that the soils were contaminated by metals and pollution was mainly focused on TS. The geoaccumulation index (*I*_geo_) and pollution indexes (*PI*) implied that there was a slight increase in the concentrations of Cd, Cu, Hg, Ni, and Pb, but the metal pollution caused by industrial activities was less severe, and elements of As and Cr exhibited non-pollution level. The risk assessment results suggested that there was a potential health risk associated with As and Cr exposure for residents because the carcinogenic risks of As and Cr via corresponding exposure pathways exceeded the safety limit of 10^−6^ (the acceptable level of carcinogenic risk for humans). Furthermore, oral ingestion and inhalation of soil particles are the main exposure pathways for As and Cr to enter the human body*.* This study may provide basic information of metal pollution control and human health protection in the vicinity of industrial regions.

## 1. Introduction

Soil is a fundamental and important natural resource and is vital to human survival. In recent years, metal pollution of soils has posed a serious threat to environmental ecosystems and human health [1,2]. This issue has received increasing attention because of the bioaccumulation, persistence, and toxicity of metals [3,4]. The atmospheric deposition of dust and aerosol, vehicle emissions, and various industrial activities are important sources of soil metal pollution [5,6]. Previous studies have reported that anthropogenic activities related to urbanization and industrialization (e.g., mining and smelting, plating, and battery manufacturing) have introduced large quantities of inorganic contaminants into the ecosystem [7,8]. Moreover, metals can be transported long distances and transferred to soils by atmospheric deposition, wastewater, and the discharge of solid wastes containing metals [9,10]. Thus, soils around industrial sites are particularly prone to act as sinks for metals.

The degree of soil metal contamination can pose a hidden danger to human health via many different ways (e.g., the oral ingestion pathway, dermal contact pathway, and inhalation pathway) [11,12]. Many studies have shown that exposure toxicity to these pollutants is influenced by several factors, including the route of exposure, absorption, metabolism and distribution in the human body [13,14]. Furthermore, a person’s age is also a significant factor that should be given more consideration. Compared with adults, children and infants are more likely to be affected because of their behavioral characteristics (e.g., outdoor activities, mouthing non-food objects, and sucking their hands or fingers) and are at greater risk of exposure to contaminants in soils [13,15,16]. Previous research has shown that food and vegetables are usually the main exposure pathways of metals in agricultural soils to the human body, due to high bioaccumulation of some toxic elements in crops [17,18]. However, for industrial soils, oral ingestion of soil particles plays an important role in exposing humans to metals [12,19]. Thus, it is more appropriate to evaluate the human health risk posed by metals in industrial soils based on direct exposure pathways because there are significant differences in the bioaccessibility of toxic elements via different exposure routes [20,21].

Dongguan, located in the Pearl River Delta in China, has undergone rapid industrialization and urbanization for the past 20 years. In recent years, high levels of persistent organic pollutants (e.g., polybrominated diphenyl ethers (PBDEs) and polycyclic aromatic hydrocarbons (PAHs)) in the sediment, dust, and air have been reported in this region [22,23]. Additionally, a large number of companies, such as hardware, mechanical, electronic and electrical, and electroplating plants, have settled in Dongguan. Despite the economic benefits, many factories discharge a large amount of metals to the soil due to industrial activities; this had led to elevated levels of metals in soils in the past several decades [24,25]. It is noteworthy that none of these laws and regulations emphasizes remediation of the contaminated soil around industrial sites; therefore, the pollutants remaining in the vicinity of industrial sites can still pose potential hazards to the surrounding environments and human health. In addition, most reports have focused on the anthropogenic influence on the surface ecological environment, agricultural soils, gardening, and market gardening in and around industrial areas [26,27,28,29]. However, few studies have been conducted on the impacts of metals in the vicinity of industrial sites. Therefore, a detailed risk assessment of metal contamination on the surrounding soil environments and inhabitants is significant for the long-term improvement of public health.

This study was conducted in Dongguan, which is one of the largest electronic manufacturing centers in the world and the urban soil has been affected intensively by human activities. The goals of this study were: (1) investigate the levels of arsenic (As), cadmium (Cd), chromium (Cr), copper (Cu), mercury (Hg), nickel (Ni), lead (Pb), and zinc (Zn) in urban soils around industrial sites of Dongguan; (2) determine the geoaccumulation index (*I*_geo_) and pollution indexes (*PI*) of metals in topsoil from the vicinity of 43 industrial sites in Dongguan; and (3) evaluate the health risks (carcinogenic and non-carcinogenic risks) to the local residents by different exposure pathways (oral ingestion, dermal and inhalation). The detailed study on the environmental quality and potential risk of urban soil around industrial regions is highly needed for the sustainable development of Dongguan. Therefore, the results of this study are useful for pollution control and risk management of metals in similar areas.

## 2. Materials and Methods

### 2.1. Study Area and Sample Collection

Dongguan (22°39′–23°09′ N, 113°31′–114°15′ E) is an industrial city situated in southeast Guangdong Province, China, which has become known as “the world’s workshop”. It has a population of approximately 12 million inhabitants in an area of 2465 km^2^. Dongguan has a typically subtropical monsoon climate with an annual average temperature and precipitation of 23.1 °C and 1820 mm, respectively. The city consists of 32 towns and districts, and the topography inclines from the southeast to the northwest. The soil parent materials in the investigated area are mainly river alluvial deposits. The soil types in Dongguan are mainly lateritic red soils (ferralsols), paddy soils (anthrosols), vegetable garden soils (anthrosols).

**Figure 1:**
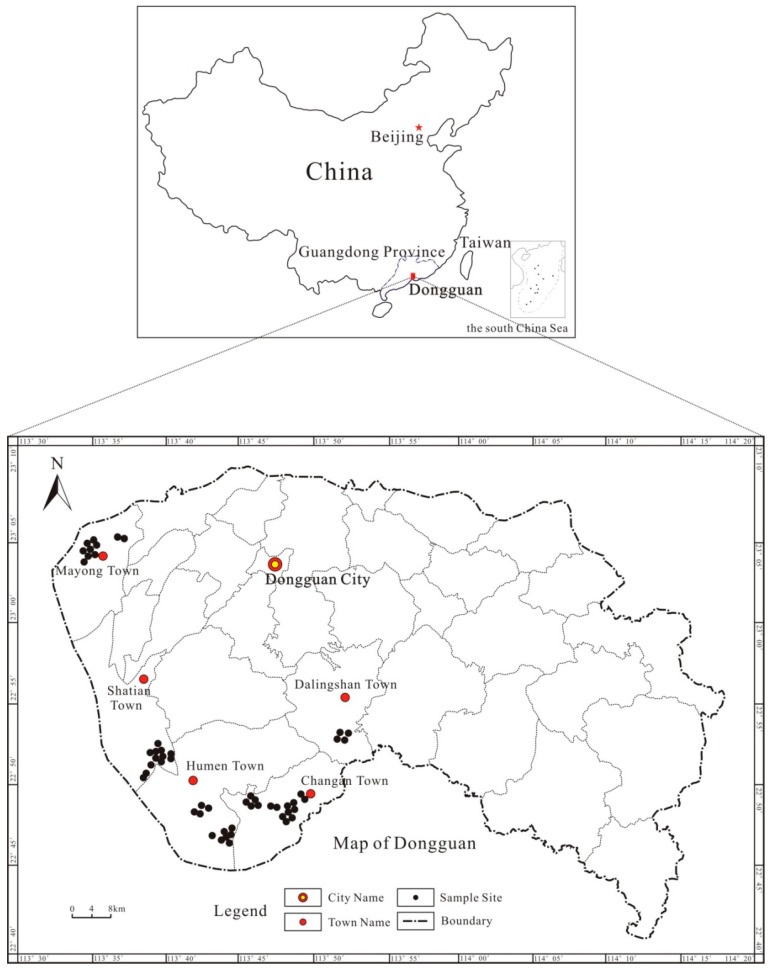
The location of the study area and distribution of sampling sites.

The locations of a total of 53 sampling sites (Changan town, Humen town, Shatian town, Machong town, and Dalingshan town) are shown on the map (Figure 1). Two types of samples were collected from each site at a depth of 0–20 cm (topsoil, TS) and 20–50 cm (shallow soil, SS). Each soil sample was a mixture of five subsamples. A total of 106 samples were collected using a stainless steel shovel (plant tissues and stones were removed), and the samples were placed into sealed Kraft packages and immediately transported to the laboratory and stored at −20 °C until ready for further analysis.

### 2.2. Chemical Analysis

All of the soil samples were air-dried in the laboratory. After drying, the samples were finely powdered using an agate mortar and passed through a 100-mesh (d < 0.154 mm) nylon sieve and then thoroughly mixed and homogenized prior to analysis for heavy metals. For the determination of the total concentration of soil metals, including Cd, Cr, Cu, Ni, Pb, and Zn, an inductively coupled plasma mass spectrometry (ICP-MS, Agilent 7700X, Agilent Technologies, Santa Clara, CA, USA) was used. For each sample, 0.2 g of soil was digested with mixed concentrated acids (HF/HNO_3_/HCl = 5:5:2), heated step-wise in a microwave oven, and the digestion solution was filtered and then diluted to 10 mL with deionized water before analysis. In addition, the total concentrations of As and Hg were measured by atomic fluorescence spectroscopy (AFS) after the soil samples were digested with aqua regia (HCl/HNO_3_, 3:1 v/v) in a 95 °C water bath for 2 h. The quality assurance and quality control (QA/QC) was conducted by using reagent blanks, replicates, and standard reference materials (GBW07403, GBW07407, and GBW07429). The recovery rates of the eight heavy metals in the soils were reasonably good (90%–121%).

### 2.3. Geoaccumulation Index (I_geo_)

The pollution level for a single element was assessed by using the geoaccumulation index (*I*_geo_) [30]. The formula for calculating of *I*_geo_ was as follows:

(1) $$I_{geo}=\log_{2}\left[\frac{C_{Sample}}{\left(1.5\times C_{Background}\right)}\right]$$

where *C_Sample_* is the concentration of the element in sample and *C_Background_* is the concentration of the element in the background, which was taken from the geochemical background values of soils in Guangdong Province, China [31]. The geochemical background value is 8.9 mg·kg^−1^ for As, 0.056 mg·kg^−1^ for Cd, 50.5 mg·kg^−1^ for Cr, 17.0 mg·kg^−1^ for Cu, 0.078 mg·kg^−1^ for Hg, 14.4 mg·kg^−1^ for Ni, 36.0 mg·kg^−1^ for Pb, and 47.3 mg·kg^−1^ for Zn. The factor 1.5 was introduced to minimize the effect of possible variations in the background values due to lithological variations. The *I*_geo_ consists of seven grades ranging from 0 to 6 (uncontaminated to extremely contaminated). The description of the *I*_geo_ classes is supplied in Table 1.

### 2.4. Pollution Indexes (PI)

Pollution indexes (*PI*) of heavy metals were used to assess the degree of metal contamination in the topsoil around the industrial areas of Dongguan. The *PI* was calculated using the soil environment quality standards of China. The *PI* was defined as follows:

(2) $$PI=\frac{C_{n}}{S_{n}}$$

where *PI* is the pollution index of the element *n*, *C_n_* is the measured concentration of the element *n* in soils (mg·kg^−1^), and *S_n_* is the geochemical background concentration of element *n* (mg·kg^−1^). The degree of heavy metal contamination in the soils can be classified into the following categories: non-pollution (*PI* ≤ 1); low level pollution (1 < *PI* ≤ 2); moderate level pollution (2 < *PI* ≤ 3); and high level pollution (*PI* > 3).

### 2.5. Human Exposureand the Health Risk Assessment Model

The concentration of metals in the soils were used to inform an exposure assessment and risk characterization for the local population according to the methodology described by the Chinese technical guidelines for risk assessment of contaminated sites [32]. The exposure of humans to heavy metals in industrial soils can occur via three main pathways such as oral ingestion, dermal contact, and inhalation of soil particles. Three routes of exposure were considered for carcinogenic and non-carcinogenic effects of trace elements present in topsoil around the industrial sites of Dongguan. The exposure dose can be estimated by using Equations (3)–(8). 

The exposure dose for carcinogenic effects was considered the lifetime (childhood and adulthood) exposure, while the exposure dose for non-carcinogenic effects was only considered for the childhood exposure. The definition and reference values for some parameters are listed in Table 2.

Exposure doses for carcinogenic effects (in a lifetime):

(3) $$OISEA_{ca}=\frac{\left(\frac{OSIR_{c}\times ED_{c}\times EF_{c}}{BW_{c}}+\frac{OSIR_{a}\times ED_{a}\times EF_{a}}{BW_{a}}\right)\times ABS_{o}}{AT_{ca}}\times 10^{-6}$$

(4) $$\begin{array}{l} DCSER_{ca}=\frac{SAE_{c}\times SSAR_{c}\times EF_{c}\times ED_{c}\times E_{v}\times ABS_{d}}{BW_{c}\times AT_{ca}}\times 10^{-6}\\ +\frac{SAE_{a}\times SSAR_{a}\times EF_{a}\times ED_{a}\times E_{v}\times ABS_{d}}{BW_{a}\times AT_{ca}}\times 10^{-6} \end{array}$$

(5) $$\begin{array}{l} PISER_{ca}=\frac{PM_{10}\times DAIR_{c}\times ED_{c}\times PIAF\times \left(fspo\times EFO_{c}+fspi\times EFI_{c}\right)}{BW_{c}\times AT_{ca}}\times 10^{-6}\\ +\frac{PM_{10}\times DAIR_{a}\times ED_{a}\times PIAF\times \left(fspo\times EFO_{a}+fspi\times EFI_{a}\right)}{BW_{a}\times AT_{ca}}\times 10^{-6} \end{array}$$

Exposure dose for non-carcinogenic effect (in childhood only):

(6) $$OISER_{nc}=\frac{OSIR_{c}\times ED_{c}\times EF_{c}\times ABS_{o}}{BW_{c}\times AT_{nc}}\times 10^{-6}$$

(7) $$DCSER_{nc}=\frac{SAE_{c}\times SSAR_{c}\times EF_{c}\times ED_{c}\times E_{v}\times ABS_{d}}{BW_{c}\times AT_{nc}}\times 10^{-6}$$

(8) $$PISER_{nc}=\frac{PM_{10}\times DAIR_{c}\times ED_{c}\times PIAF\times \left(fspo\times EFO_{c}+fspi\times EFI_{c}\right)}{BW_{c}\times AT_{nc}}\times 10^{-6}$$

The carcinogenic risk (*CR*) was calculated to evaluate the risk of each element in the soil via the corresponding exposure pathway. The *CR* was multiplied by the concentration of the surface soils (*C_sur_*) and the carcinogenic slope factor (*SF*). The *SF* for carcinogenic elements is shown in Table 3. The *CR* was calculated using Equations (9)–(11):

(9) $$CR_{ois}=OISER_{ca}\times C_{sur}\times SF_{o}$$

(10) $$CR_{dcs}=DCSER_{ca}\times C_{sur}\times SF_{d}$$

(11) $$CR_{pis}=PISER_{ca}\times C_{sur}\times SF_{i}$$

The exposure dose of each element for a non-carcinogenic effect can be determined by calculating the non-carcinogenic hazard quotient (*HQ*). The *HQ* was calculated using Equations (12)–(14). If the value of *CR* is higher than 10^−6^, it means there is a probability of a carcinogenic risk, whereas if the *HQ* exceeds 1.0, it is likely that there will be adverse effects to human health [33]. The *HI* was calculated as the sum of *HQ*s from the different pathways. *HI* was calculated using Equation (15). The reference dose (*RfD*) for non-carcinogenic metals is shown in Table 3.

(12) $$HQ_{ois}=\frac{OISER_{nc}\times C_{sur}}{RfD_{o}\times SAF}$$

(13) $$HQ_{dcs}=\frac{DCSER_{nc}\times C_{sur}}{RfD_{d}\times SAF}$$

(14) $$HQ_{pis}=\frac{PISER_{nc}\times C_{sur}}{RfD_{i}\times SAF}$$

(15) $$HI=HQ_{ois}+HQ_{dcs}+HQ_{pis}$$

### 2.6. Statistical Analysis

Statistical analyses were conducted with SPSS 17.0 (SPSS Inc., Chicago, IL, USA) and Origin 8.5 (OriginLab, Northampton, MA, USA). An analysis of variances ANOVA (*p* < 0.05) was performed to examine the statistical significance of heavy metal concentrations among the different sampling sites. A correlation analysis was used to determine the relationship between the concentrations of all of the elements in the topsoil of the study area. The criteria for significance in the procedures was set at *p* < 0.05 (significant) and *p* < 0.01 (highly significant).

## 3. Results and Discussion

### 3.1. Heavy Metal Concentration in the Soil

**Figure 2:**
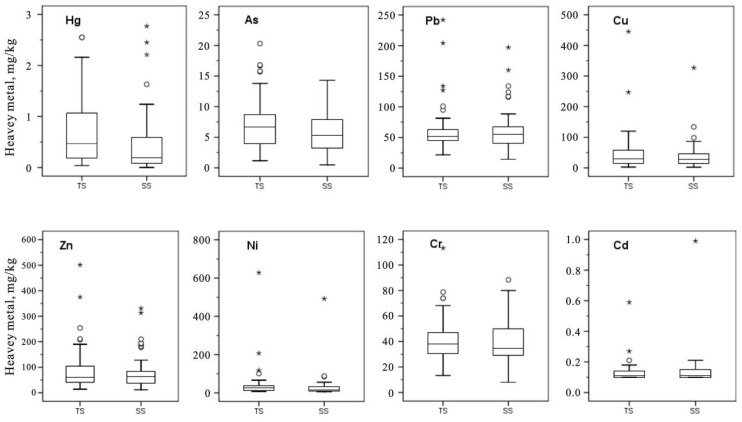
Heavy metal concentrations of soil from the vicinity of industrial sites in Dongguan. TS: topsoils of 0–20 cm, SS: shallow soils of 20–50 cm. The circle symbols (○) represent outliers (mild outliers), and the cross symbols (*) represent extremes (extreme outliers). The horizontal lines at the top, middle and bottom of the box plot correspond to the 75th percentile, median and 25th percentile, respectively.

The spatial distribution of heavy metals in soils from the vicinity of the industrial sites is depicted in Figure 2. The soils in this study showed distinct changes in their concentrations of heavy metals. The concentrations of eight heavy metals (As, Cd, Cr, Cu, Hg Ni, Pb, and Zn) in TS varied between 1.2 and 20.3, 0.1 and 0.6, 13.4 and 113.2, 2.7 and 445.0, 7.7 and 628.0, 21.6 and 242.0, and 13.9 and 501.0 mg·kg^−1^, respectively. The coefficient of variation (*CV*) indicates the degree of variability within the concentrations of a metal in the soil [34]. The *CV* of metals in TS for this study decreased in the order of Ni (205.68%) > Cu (142.18%) > Hg (96.26%) > Zn (96.01%) > Pb (63.59%) > As (61.76%) > Cd (55.50%) > Cr (42.19%). The large *CV* for these heavy metal concentrations suggests that considerable variability exists in the different sites, which reflects the non-homogeneous spatial distribution of heavy metal concentrations in this area. Moreover, the average concentrations of As (7.1 mg·kg^−1^), Cr (40.8 mg·kg^−1^), Cu (48.3 mg·kg^−1^), Hg (0.7 mg·kg^−1^), Ni (42.8 mg·kg^−1^), Pb (61.8 mg·kg^−1^), and Zn (92.0 mg·kg^−1^) in TS were 1.23, 1.02, 1.30, 1.49, 1.38, 1.01, and 1.15-fold higher than the corresponding mean values in SS, respectively, and Cd (0.13 mg·kg^−1^) was 1.08-fold lower than the average concentration in SS. This is because these heavy metals were grouped in TS from the vicinity of the industrial sites during the period when industrial activities were most extensive. In particular, the maximum concentrations of As (20.3 mg·kg^−1^), Cr (113.2 mg·kg^−1^), Cu (445.0 mg·kg^−1^), Ni (628.0 mg·kg^−1^), Pb (242.0 mg·kg^−1^), and Zn (501.0 mg·kg^−1^) were found in TS. Whereas the maximum concentrations of Cd (1.0 mg·kg^−1^) and Hg (2.8 mg·kg^−1^) were analyzed onlyin SS. Therefore, the distribution characteristics of the heavy metals again confirmed that the topsoil surrounding the industrial regions were strongly contaminated by heavy metals.

Due to the lack of appropriate comparable information in SS in similar areas, only heavy metal contaminations in TS from the other study areas were compared with our data in this study. The heavy metal concentrations in TS collected from the vicinity of the industrial area in Dongguan are compared with data reported for other areas in China, and reference soil guideline values are listed in Table 4. Compared with background values, the mean concentrations of Cd, Cu, Hg, Ni, Pb, and Zn, greatly exceeded the geochemical background values of Guangdong Province [31]. This demonstrated that the topsoils from the vicinity of the industrial sites of Dongguan were heavily polluted. However, the mean concentrations of the metals, except Hg and Ni, were below the Grade II guideline values [35]. By comparison with the Dutch standards [36], the mean concentrations of all of the metals were below the Dutch intervention values, whereas only the mean concentrations of Hg, Cu, and Ni exceeded the Dutch target values. In addition, the present Cu (48.3 mg·kg^−1^) and Pb (61.8 mg·kg^−1^) levels in the study area were higher than Yan’an (27.31 mg·kg^−1^; 23.97 mg·kg^−1^), Weinan (20.88 mg·kg^−1^; 46.71 mg·kg^−1^), and Lipu (40.77 mg·kg^−1^; 50.11 mg·kg^−1^) [37,38,39]. However, the mean Cr concentration was lower than threeother cities, and the mean concentrations of Cd (0.13 mg·kg^−1^) and Ni (42.8 mg·kg^−1^) were lower than Lipu (0.19 mg·kg^−1^; 53.65 mg·kg^−1^) [39]. In addition, the average values of Ni (42.8 mg·kg^−1^) and Zn (92.0 mg·kg^−1^) were higher than both Yan’an (38.01 mg·kg^−1^; 82.15 mg·kg^−1^) and Weinan (25.43 mg·kg^−1^; 71.56 mg·kg^−1^) [37,38]. When compared with the three other cities in China, the relatively higher levels of heavy metals in topsoil around the industrial sites of Dongguan could be due to industrial emissions in this region during the last decades.

A correlation of heavy metals was applied to analyze the sources and pathways among the heavy metals [40]. The Spearman’s correlation coefficients are shown in Table 5. The results demonstrate that the elemental pairs Pb-Cu (0.523), Pb-Zn (0.395), Cu-Zn (0.637), Cu-Ni (0.520), and Zn-Ni (0.455) had a significantly positive correlation at the *p* < 0.01 significance level andPb and Ni (0.348) at the *p* < 0.05 significance level, which revealed that these elements most likely originate from some common sources.

Many previous reports have confirmed that elements, such as As, Cd, Cu, Hg, Ni, Pb, and Zn in the soils, originate mainly from anthropogenic activities [9,11,41]. Consequently, we could further infer that there are some relationships between the sources of most of the toxic elements in this study and anthropogenic inputs.

### 3.2. The I_geo_ of Heavy Metals

**Figure 3:**
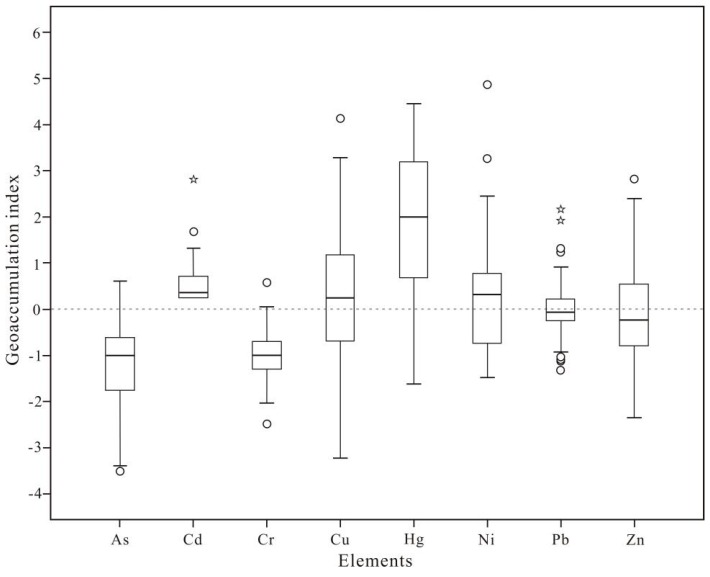
Box plot of *I*_geo_ for heavy metals in topsoils around industrial sites in Dongguan. The *circles* represent outliers (mild outliers), and the *stars* represent extremes (extreme outliers). The *horizontal* lines at the top, *middle* and *bottom* of the box plot correspond to the 75th percentile, median and 25th percentile, respectively.

The contamination status of the heavy metals in the research area was evaluated by the *I*_geo_ (Figure 3). The *I*_geo_ ranged from −3.51 to 0.61 for As, 0.25 to 2.81 for Cd, −2.50 to 0.58 for Cr, −3.22 to 4.13 for Cu, −1.62 to 4.45 for Hg, −1.48 to 4.86 for Ni, −1.32 to 2.16 for Pb, and −2.35 to 2.82 for Zn. The severity of the pollution gauged by the mean of *I*_geo_ decreased in the order of Hg (1.86) > Cd (0.55) > Ni (0.22) > Cu (0.19) > Pb (0.01) > Zn (−0.06) > Cr (−1.00) > As (−1.21). The mean *I*_geo_ of Hg showed a moderately contaminated result, whereas a slightly to moderately contaminated status was found for Cd, Cu, Ni, and Pb, which indicates that the means of *I*_geo_ varied between 0 to 1. The mean *I*_geo_ of As, Cr, and Zn were beyond 0 as well, indicating a largely uncontaminated status. In addition, compared with the background conditions, the *I*_geo_ values for Cd, Cu, Hg, Ni, and Pb manifested that there was a slight increase in the concentrations of these elements, and the heavy metal pollution caused by industrial activities was less severe, whereas no such increases are observed in the concentrations of As, Cr, and Zn.

The relocation of industrial manufacturing from developed countries to Dongguan has promoted the urbanization and industrialization in this area since China’s open door policy in 1978 [42]. However, the rapid economic growth over the past two decades in this region has brought significant environmental problems, including encroachment on agricultural land, soil erosion, and pollution. Previous studies conducted in Dongguan found that the arable land decreased from 30,816 ha in 2004 to 24,800 ha in 2012, whereas concentrations of heavy metal in soils from urbanized areas of Dongguan increased by 28.6% for Cd, 33.0% for Cu, 16.2% for Ni, and 55.9% for Zn [24,43,44]. Many researchers have reported that Cd, Cu, and Zn were the major contaminants in topsoils surrounding industrial plants [10,12,44]. These results were very similar to the findings in this research. Therefore, close attention must be paid to pollution by toxic metals in the vicinity of industrial sites because of the human activities are still taking place in these fields. 

### 3.3. Pollution Indexes of Heavy Metals

A pollution evaluation was conducted for eight heavy metals, including As, Cd, Cr, Cu, Hg, Ni, Pb, and Zn, based on the geochemical background values of Guangdong Province [31]. The statistical results of *PI* for each metal are shown in Table 6. Overall, the mean *PI* for all of the metals were in descending order of Hg (8.89) > Ni (2.97) > Cu (2.84) > Cd (2.36) > Zn (1.95) > Pb (1.72) > Cr (0.81) > As (0.80). The *PI* values of Cd, Cu, Hg, Ni, Pb, and Zn are much higher, ranging from 1.79 to 10.54, 0.16 to 26.21, 0.49 to 32.70, 0.54 to 43.63, 0.60 to 6.71, and 0.29 to 10.59. The mean *PI* of Hg pointed to a high level of pollution, while the mean *PI* obtained for Cd, Cu, Ni, Pb, and Zn indicate low to moderate levels of pollution. As and Cr exhibited lower values, ranging from 0.13 to 2.29 and 0.26 to 2.24, respectively, this indicated that the topsoil in the area were non-polluted by As and Cr. Indeed, most of the samples had non-pollution level *PI* values for As and Cr, and only 13 and 10 samples had *PI* levels indicating low to moderate level pollution, respectively. However, high *PI* values (higher than 1) were observed in 100.0% of the samples for Cd, 69.8% for Cu, 96.2% for Hg, 69.8% for Ni, 86.8% for Pb, and 66.0% for Zn. These findings indicate that according to the geochemical background values of Guangdong Province, the topsoils in the vicinity of the industrial sites of Dongguan are contaminated by Cd, Cu, Hg, Ni, Pb, and Zn.

**Figure 4:**
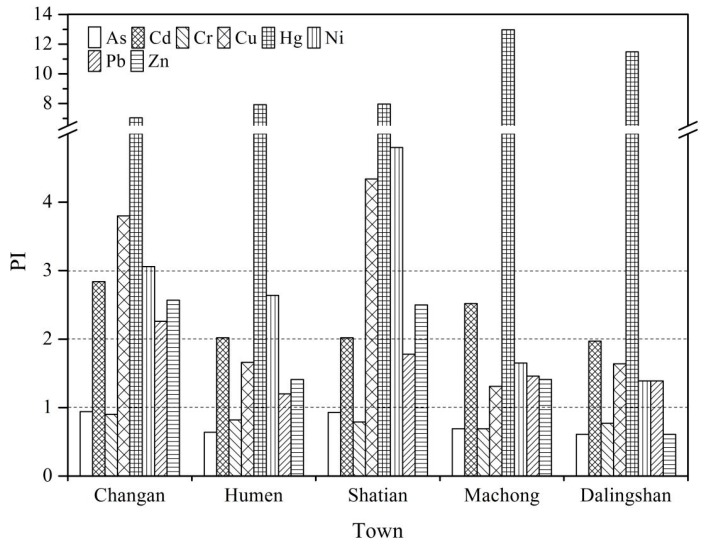
The mean pollution index (*PI*) of heavy metals in topsoils in different towns of Dongguan.

As illustrated in Figure 4 and in comparison with other towns, the *PI* values were generally higher in Changan and Shatian, indicating the presence of a relatively serious heavy metal pollution problem in Changan and Shatian. The mean *PI* value of Hg in the five towns was higher than 3, which indicated high Hg pollution of the soils. This might partially explain that the soil properties are an important factor for the ability to bind and accumulate Hg. For soil Hg, previous research on Guangdong soil profiles showed that there were significant positive correlations with only soil organic matter (SOM) contents, whereas soil properties play a lesser role in the concentration and distribution of soil Hg [26]. Another reason for this phenomenon might be that anthropogenic input or atmospheric deposition was probably the major contributor for the enrichment of soil Hg. Previous studies have also confirmed that anthropogenic Hg was the primary source of soil Hg in Guangdong [45]. In contrast, the mean *PI* values of As and Cr in five towns were lower than 1, showing that there was no obvious pollution of As and Cr in the soils. Moreover, As and Cr contents of the soil are derived from parent rocks and it is likely that past human activities, such as earth movement and leveling, resulted in the addition of uncontaminated soil material on top of the soil surface. 

In the towns of Humen and Machong, the PI values of Cd, Cu, Ni, Pb, and Zn were between 1 and 3, which indicated low to moderate pollution. Dalingshan was predominantly polluted with Cd, Cu, Ni, and Pb with PI values of 1.97, 1.64, 1.39, and 1.39, respectively, which indicated low levels of pollution. The rapid development of the electronics and electroplating industries in recent decades is likely the main cause for the Cd, Cu, Ni, Pb, and Zn pollution in these five towns. In addition, to accurately evaluate the risk of heavy metals to humans in topsoil around the industrial sites of Dongguan, a detailed health risk assessment should be conducted. 

### 3.4. Health Risk Assessment

**Figure 5:**
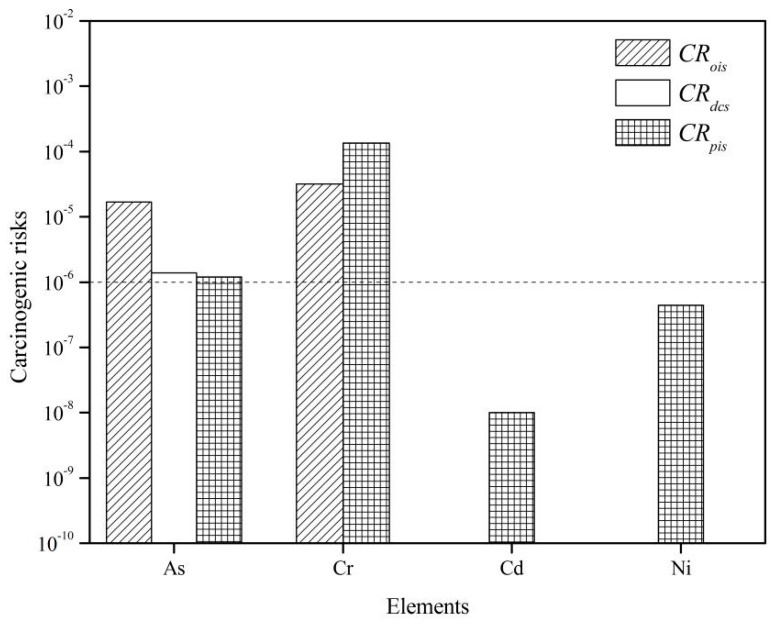
Carcinogenic risks of heavy metals in topsoils around industrial sites in Dongguan.

As shown in Figure 5, the carcinogenic risk values of As via each exposure pathway are 1.68 × 10^−5^ for *CR_ois_*, 1.39 × 10^−6^ for *CR_dcs_*, and 1.20 × 10^−6^ for *CR_pis_*. The *CR* values of As via the three exposure pathways exceed the safety limit 10^−6^, especially for the exposure of As via oral ingestion of soil particles, which has the highest *CR* value and suggests that there may be a potential health risk associated with As exposure to the local residents of Dongguan. Several studies have observed similar results [46,47,48,49]. In addition, the *CR* values of Cr via oral ingestion (*CR_ois_* 3.20 × 10^−5^) and inhalation (*CR_pis_* 1.34 × 10^−4^) were also higher than the safety limit of 10^−6^. This indicates that oral ingestion and inhalation of Cr through soil particles in the vicinity of industrial sites could result in an increased risk of cancer for humans. Other reports have also identified an increased carcinogenic risk of Cr present in soil particles [48,50]. 

**Figure 6:**
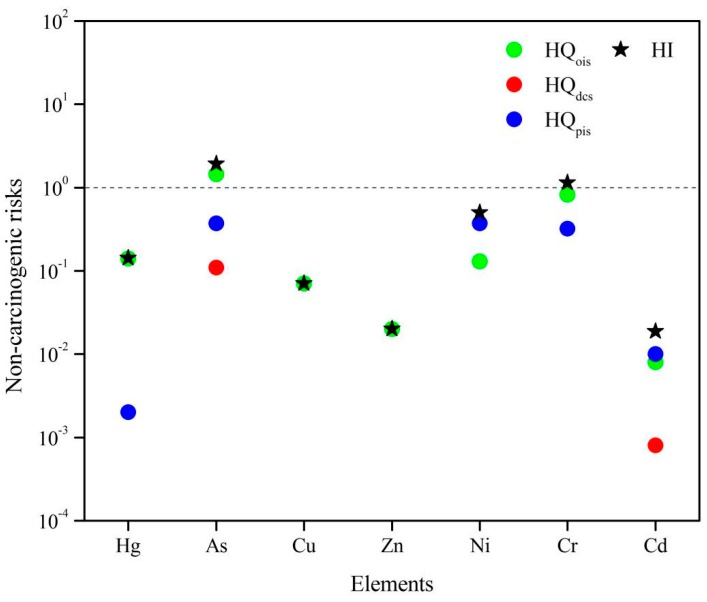
Non-carcinogenic risks of heavy metals in topsoils around industrial sites in Dongguan.

In contrast, *CR_pis_* of Cd (0.01 × 10^−6^) and Ni (0.44 × 10^−6^) are both at acceptable levels of carcinogenic risk for humans. The *HQ*s derived from various exposure pathways are depicted in Figure 6. The *HI* values of heavy metals for children decreased in the order of As (1.920) > Cr (1.140) > Ni (0.500) > Hg (0.142) > Cu (0.070) > Zn (0.020) > Cd (0.019). The *HI* values of element As and Cr were higher than 1.0, indicating that there was a potential non-carcinogenic risk to children, whereas the elements Ni, Hg, Cu, Zn, and Cd in soil samples with *HI* values were lower than 1.0, showing that there was no non-carcinogenic risk for children. Furthermore, by comparing the *HQ* values for children, we concluded that the oral ingestion of soil particles is the main exposure pathway of heavy metals to children. This result is consistent with previous studies [41,51]. In summary, the non-carcinogenic risk of As and Cr cannot be ignored for children’s health because the *HI* values exceed 1.0, which indicates that children face more health risk due to their pica behavior and hand or finger sucking [52]. Additionally, the consumption of local vegetables and grain could present health risks to children [53].

It is important to note that most current risk assessments are still based on the total concentration of heavy metals in soil, which are appropriate for long-term risks or for worst-case scenarios and might overestimate the actual health risks [54,55]. For example, chromium toxicity is directly dependent on its valence state, and Cr(VI) has a higher toxicity to biota than Cr(III). Previous studies have confirmed that Cr(VI) is a human carcinogen [19,34]. In addition, Luo et al. introduced the idea of bioaccessibility of heavy metals in soil to the assessment of human health risk [35]. Metal bioaccessibility is the fraction that is soluble in the gastrointestinal tract and available for absorption. Although information on the bioaccessibility of heavy metals in this study is limited, we can still provide an accurate assessment of the risk status of soils by using various other parameters. It may be inferred that As and Cr in topsoil around the industrial areas of Dongguan are the main contaminants that pose both carcinogenic and non-carcinogenic risks to human health. The oral ingestion of soil particles is the main exposure pathway for As and Cr to enter the human body. Hg, Cu, Zn, and Cd may pose a public health risk because of the concentrations of these elements in topsoil around the industrial areas of Dongguan. 

In addition, in this study, only heavy metal pollutants were selected for the risk assessment of soils from the vicinity of the industrial sites. However, organic contaminants, such as polychlorinated dibenzo-*p*-dioxins/furans (PCDD/Fs) [56], dichlorodiphenyltrichloroethanes (DDTs) [57], halogenated flame retardants (HFRs) [22], and perfluoroalkyl acids (PFAAs) [58] may also be present in these soils. Thus, the actual pollution level of soils around industrial sites might be higher than those indicated by the above results. Thus, efforts need to be taken immediately to control the emission of pollutants and remediate contaminated soils in similar areas.

## 4. Conclusions

The concentration, distribution, pollution, and health risk assessment of heavy metals in soils around industrial sites of Dongguan were thoroughly investigated in this study. The pollution levels in TS from the study area were higher than those in SS, and elements As, Cr, Cu, Hg, Ni, Pb, and Zn were mainly grouped in the TS. Compared with the Guangdong background values of these elements in the soil, concentrations of Cd, Cu, Hg, Ni, Pb, and Zn were elevated in the vicinity of industrial sites in Dongguan. The Spearman correlation analysis showed that Cu, Ni, Pb, and Zn originated from common anthropogenic sources. The calculated *I_geo_* of the analyzed heavy metals indicate that there was a slight increase in the concentrations of Cd, Cu, Hg, Ni, and Pb. Higher mean *PI* values for Cd, Cu, Hg, Ni, Pb, and Zn in this study indicate that there is low to high level pollution, which mainly originates from industrial emissions. The *I_geo_* and *PI* values of As and Cr were low, indicating that As and Cr were practically unpolluted in this area.

A health risk assessment method based on the Chinese technical guidelines for risk assessment of contaminated sites was used to assess human exposure to heavy metals from soils in the research region. The potentially higher carcinogenic risks mainly resulted from As and Cr via the oral ingestion and inhalation exposure pathways. In addition, the non-carcinogenic risk values of As (1.920 for *HI*) and Cr (1.140 for *HI*) for children is slightly higher than the threshold value (1.0), indicating that children are facing a slight threat from As and Cr. The main exposure pathway of heavy metals for children is the oral ingestion of soil particles. These findings indicate that more attention should be focused on heavy metal contamination to reduce health risks of residents living in the vicinity of industrial sites in Dongguan. In this regard, this study may also guide policy decisions aimed at protecting the local environment and human health, such as polluted soils’ restoration and management, and related pollution industries’ remediation.

## Figures and Tables

**Table 1:** The *I*_geo_ and classification of pollution degree.

*I*_geo_ Class	*I*_geo_ Value	Pollution Degree
0	*I*_geo_ ≤ 0	Uncontaminated
1	0 < *I*_geo_ ≤ 1	Slightly to moderately contaminated
2	1 < *I*_geo_ ≤ 2	Moderately contaminated
3	2 < *I*_geo_ ≤ 3	Moderately to heavily contaminated
4	3 < *I*_geo_ ≤ 4	Heavily contaminated
5	4 < *I*_geo_ ≤ 5	Heavily to extremely contaminated
6	*I*_geo_ > 5	Extremely contaminated

**Table 2:** Definition and reference value of some parameters for health risk assessment of heavy metals in soils [32].

Symbols	Units	Definition	Adult Value	Child Value
ABS_o_, ABS_d_		Absorption factor of oral ingestion and dermal contact of soil particles, respectively		
AT_ca_, AT_nc_	day	Average time for carcinogenic and non-carcinogenic effects, respectively	26,280; 2190	26,280; 2190
BW_a_, BW_c_	kg	Average body weight of adults and children, respectively	56.8	15.9
OISER_ca_, DCSER_ca_, PISER_ca_	kg·kg^−^^1^·day^−^^1^	Chronic daily intake or exposure dose through oral ingestion, dermal contact and inhalation of soil particles, respectively		
CR_ois_, CR_dcs_, CR_pis_		Cancer risk of heavy metal through oral ingestion, dermal contact and inhalation of soil particles, respectively		
DAIR_a_, DAIR_c_	m^3^·day^−^^1^	Daily air inhalation rate of adults and children, respectively	14.5	7.5
ED_a_, ED_c_	a	Exposure duration of adults and children, respectively	24	6
EF_a_, EF_c_	day·a^−^^1^	Exposure frequency of adults and children, respectively	350	350
EFI_a_, EFI_c_	day·a^−^^1^	Indoor exposure frequency of adults and children, respectively	262.5	262.5
EFO_a_, EFO_c_	day·a^−^^1^	Outdoor exposure frequency of adults and children, respectively	87.5	87.5
E_v_	day^−^^1^	Daily exposure frequency of dermal contact event	1	1
fspi, fspo		Fraction of soil-borne particles in indoor and outdoor air, respectively	0.8; 0.5	0.8; 0.5
HI		Hazard index of heavy metal		
HQ_ois_, HQ_dcs_, HQ_pis_		Hazard quotient of heavy metal through oral ingestion, dermal contact and inhalation of soil particles, respectively		
OSIR_a_, OSIR_c_	mg·day^−^^1^	Daily oral ingestion rate of soils of adults and children, respectively	100	200
PIAF		Retention fraction of inhaled particulates in body	0.75	0.75
PM_10_	mg·m^−^^3^	Content of inhalable particulates in ambient air	0.15	0.15
RfD_o_, RfD_d_, RfD_i_	mg·kg^−^^1^·day^−^^1^	Reference dose of heavy metal through oral ingestion, dermal contact and inhalation of soil particles, respectively		
SAE_a_, SAE_c_	cm^2^	Surface area of exposed skin for adults and children, respectively	5075	2448
SAF		Soil allocation factor	0.20	0.20
SF_o_, SF_d_, SF_i_	(mg·kg^−^^1^·day^−^^1^)^−^^1^	Cancer slope factor of heavy metal via oral ingestion, dermal contact and inhalation of soil particles, respectively		
SSAR_a_, SSAR_c_	mg·cm^−^^2^	Adherence rate of soil on skin for adults and children, respectively	0.07	0.2

**Table 3:** *SF* for carcinogenic metals and *RfD* for non-carcinogenic metals [32].

Elements	*SF*/(mg/kg·d)^−1^			*RfD*/mg/(kg·d)		
	*SF_o_*	*SF_d_*	*SF_i_*	*RfD_o_*	*RfD_d_*	*RfD_i_*
As	1.50	1.50	16.80	3 × 10^−4^	3 × 10^−4^	3.38 × 10^−6^
Cd	-	-	7.05	0.001	2.5 × 10^−5^	2.55 × 10^−6^
Cr	0.50	20.00	329.00	0.003	7.5 × 10^−5^	2.55 × 10^−5^
Cu	-	-	-	0.04	0.04	-
Hg	-	-	-	3 × 10^−4^	2.1 × 10^−5^	7.66 × 10^−5^
Ni	-	-	1.02	0.02	8 × 10^−4^	2.3 × 10^−5^
Zn	-	-	-	0.3	0.3	-

**Table 4:** Concentrations of heavy metals in topsoils from the vicinity of industrial areas and related soil quality standards.

Places		Depth	Hg	As	Pb	Cu	Zn	Ni	Cr	Cd
Dongguan	This study	0–20 cm	0.7	7.1	61.8	48.3	92.0	42.8	40.8	0.13
Yan’an	reference [37]	0–20 cm	-	-	23.97	27.31	82.15	38.01	73.88	0.11
Weinan	reference [38]	0–15 cm	-	8.49	46.71	20.88	71.56	25.43	96.99	-
Lipu	reference [39]	0–15 cm	-	-	50.11	40.77	-	53.65	46.98	0.19
Chinese [35]	I	-	0.15	15	35	35	100	40	90	0.2
	II (pH < 6.5)	-	0.3	40	250	50	200	40	150	0.3
	III	-	1.5	40	500	400	500	200	300	1
Guangdong background values [31]		-	0.078	8.9	36.0	17.0	47.3	14.4	50.5	0.056
Dutch [36]	Target values	-	0.3	29	85	36	140	35	100	0.8
	Intervention values	-	10	55	530	190	720	210	380	12

I. the limits for protecting ecosystem; II. the maximum allowable concentrations of metals in agriculture soil of China; III. the upper limit values for regular growing of plants.

**Table 5:** Correlation coefficient among different metals in topsoils around industrial sites in Dongguan.

Elements	Hg	As	Pb	Cu	Zn	Ni	Cr
As	−0.069	1					
Pb	0.221	0.257	1				
Cu	0.199	0.101	0.523 **	1			
Zn	0.119	−0.043	0.395 **	0.637 **	1		
Ni	0.264	−0.234	0.348 *	0.520 **	0.455 **	1	
Cr	0.089	0.043	0.183	0.033	−0.089	0.185	1
Cd	0.245	−0.011	0.062	0.076	0.087	0.261	0.121

* Correlation is significant at the 0.05 level (2-tailed); ** Correlation is significant at the 0.01 level (2-tailed).

**Table 6:** Statistical results of pollution index (*PI*) of heavy metals in topsoils around industrial sites in Dongguan.

Elements	*PI*			Number of Samples			
	Mean	Min	Max	h.p ^a^	m.p ^a^	l.p ^a^	n.p ^a^
As	0.80	0.13	2.29	0	1	12	40
Cd	2.36	1.79	10.54	6	20	27	0
Cr	0.81	0.26	2.24	0	1	9	43
Cu	2.84	0.16	26.21	15	7	15	16
Hg	8.89	0.49	32.70	36	5	10	2
Ni	2.97	0.54	43.63	8	17	12	16
Pb	1.72	0.60	6.71	4	6	36	7
Zn	1.95	0.29	10.59	10	6	19	18

^a^ h.p: high level pollution; m.p: moderate level pollution; l.p: low level pollution; n.p: non-pollution.
